# Supplementary material for: Systemic Chemotherapy in Colorectal Peritoneal Metastases Treated with Cytoreductive Surgery: Systematic Review and Meta-Analysis
Source: Cancers (Basel). 2024 Mar 18;16(6):1182. doi: 10.3390/cancers16061182 (PMC10969605; doi:10.3390/cancers16061182)
Supplement: Supplementary file 1 [file cancers-16-01182-s001.zip › cancers-2886669-supplementary.pdf]

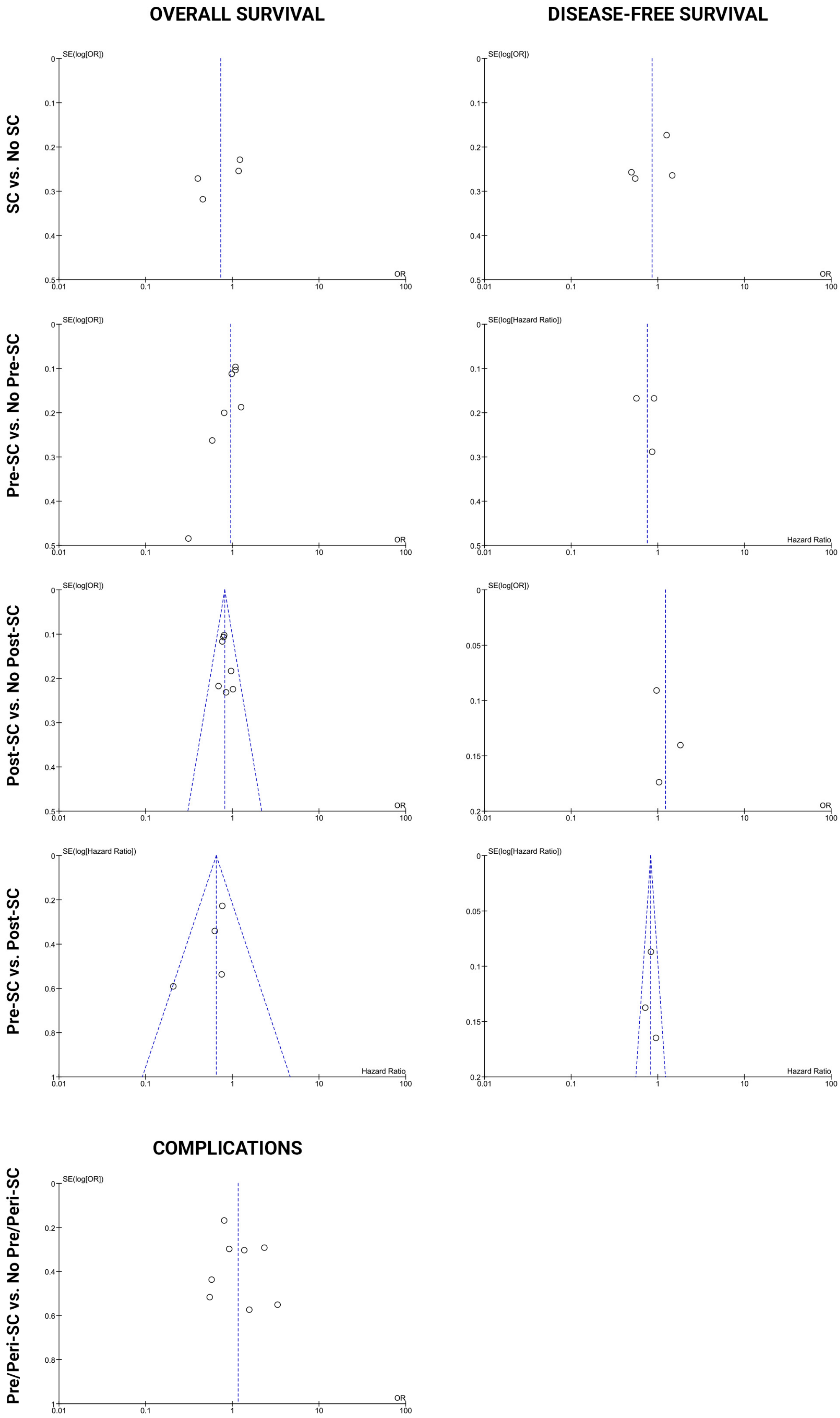

Figure S1. Funnel plots of the performed analyses.

Table S1. Studies excluded after full-text evaluation.

First Author, Year	Study design (centers n)	Study period	Country	Reason for exclusion
Shen, 2004	retrospective single center	1991-2002	USA	**CC2 = 52%**
Chua, 2011	retrospective multicentric (3)	1988-2009	Germany, Australia, USA	**CC2 = 73%**
Adachi, 2015	retrospective single center	1992-2012	Japan	**CC2 = 70%**
Glockzin, 2018	prospective multicenter (10)	2010-2014	Germany	**CC2 = 38%**
Leimnkuhler, 2019	retrospective and prospective single center	2006-2015	Netherlands	**CC2 = 33%**
Zhou, 2021	retrospective multicentric (2)	2017-2019	China	**CC2 = 40%**

Table S2. Newcastle-Ottawa Scale (NOS) for assessing the quality of studies in meta-analyses.

		COHORT STUDY													CASE CONTROL STUDY											
		**S1**	**S2**	**S3**	**S4**	**C**	**O1**	**O2**	**O3**	**Total**	**S**	**C**	**O**	**Quality**	**S1**	**S2**	**S3**	**S4**	**C**	**E1**	**E2**	**Total**	**S**	**C**	**E**	**Quality**
**1**	**Glehen, 2004**	1	1	1	1	1	1	1		**7**	4	1	2	**Good**												
**2**	**Elias, 2010**	1	1	1	1	1	1	1		**7**	4	1	2	**Good**												
**3**	**Passot, 2012**	1	1	1	1	1	1	1		**7**	4	1	2	**Good**												
**4**	**Baratti, 2014**	1	1	1	1	1	1	1		**7**	4	1	2	**Good**												
**5**	**Ceelen, 2014**	1	1	1	1	1	1	1		**7**	4	1	2	**Good**												
**6**	**Kuijpers, 2014**	1	1	1	1		1	1		**6**	4	0	2	**Fair**												
**7**	**Devilee, 2016**	1	1	1	1	1	1	1		**7**	4	1	2	**Good**												
**8**	**Maillet, 2016**	1	1	1	1	1	1	1		**7**	4	1	2	**Good**												
**9**	**van Eden, 2017**	1	1	1	1	1	1	1		**7**	4	1	2	**Good**												
**10**	**Beal, 2020**	1	1	1	1	1	1	1		**7**	4	1	2	**Good**												
**11**	**Repullo, 2021**			1	1	1	1	1		**5**	2	1	2	**Fair**												
**12**	**Rovers, 2021**														1	1	1	1	2	1	1	**8**	4	2	2	**Good**
**13**	**Hanna, 2022**	1	1	1	1	1	1	1		**7**	4	1	2	**Good**												
**14**	**Cashin, 2023**														1	1	1	1	2	1	1	**8**	4	2	2	**Good**
**15**	**Tonello, 2023**														1	1	1	1	2	1	1	**8**	4	2	2	**Good**
	**Median**									**7**	4	1	2	**Good**								**8**	4	2	2	**Good**

Good: 3-4 S and 1-2 C and 2-3 O/E.
Fair: 2 S and 1-2 C and 2-3 O/E.
Poor: 0-1 S or 0 C or 1 O/E.
Abbreviations. S: Selection; C: Comparability; O: Outcome; E: Exposure.

Table S3. Meta-regression results for severe complications sub-analysis.

	Period	Design	Target therapy	Triplet SC
**Baratti, 2014**	N	N	N	N
**Devilee, 2016**	N	N	N	N
**van Eden, 2017**	N	N	-	N
**Beal, 2020**	N	N	Y	Y
**Repullo, 2021**	Y	Y	Y	N
**Rovers, 2021**	Y	N	Y	N
**Cashin, 2023**	Y	Y	-	-
**Tonello, 2023**	Y	Y	Y	Y
**Residual heterogeneity (I^2)**	65.9%	68.3%	65.0%	49.4%
**Amount of heterogeneity accounted for (R^2)**	0%	0%	0%	29.8%
**p-value**	0.316	0.774	0.251	0.196

Period: studies with median year of patient enrollment after 2009 (year of all included studies).
Design: studies with a design with low-risk of bias (Yes: randomized controlled trials or propensity-score studies) compared to high-risk (No: retrospective series).
Target therapy: studies using target therapy in at least 50% of patients.
Triplet: studies using triplet systemic chemotherapy (FOLFOXIRI).
Y: yes, **N**: no.
